# Maternal and paternal alcohol consumption in the prenatal period and mental health and behavior of their children until adulthood

**DOI:** 10.1192/j.eurpsy.2025.10035

**Published:** 2025-05-30

**Authors:** Zuzana Mohrová, Zsófia Csajbók, Albert Kšiňan, Lenka Andrýsková, Pavla Brennan Kearns

**Affiliations:** 1 Department of Epidemiology, Second Faculty of Medicine, Charles University, Prague, Czech Republic; 2 Faculty of Humanities, Charles University, Prague, Czech Republic; 3 RECETOX, Faculty of Science, Masaryk University, Brno, Czech Republic

**Keywords:** child development, cohort study, mental health of children, parental alcohol consumption

## Abstract

**Background:**

Maternal alcohol consumption can adversely affect children’s development, but the impact of paternal drinking is less understood. We aimed to investigate whether maternal or paternal alcohol consumption during pregnancy affected children’s mental health and behavior.

**Methods:**

A total of 2,013 parent–child triads from the European Longitudinal Study of Pregnancy and Childhood cohort were used. Data on alcohol consumption was obtained from questionnaires during pregnancy and after the child’s birth. Mental health and behavior of children were assessed with Strength and Difficulties Questionnaire (SDQ). The associations were tested using linear regression, adjusting for socio-demographic and psychosocial covariates.

**Results:**

Increased maternal alcohol consumption was associated with higher total SDQ scores at ages 7, 11, and 18 years old when the outcomes were reported by mothers, but only at 11 years when reported by children. We did not observe any dose–response relationship, and the effect size did not change during the follow-up. The effects were observed across various domains of SDQ: in the emotional symptoms subscale at age 11, in the conduct problems subscale at ages 7 and 11, and in the hyperactivity/inattention subscale at age 18. Paternal alcohol consumption was not associated with SDQ.

**Conclusions:**

Maternal alcohol consumption during pregnancy is associated with long-term effects on children’s mental health and behavior, particularly when reported by mothers. No association was found between paternal alcohol consumption, suggesting that the results may stem from biological effects of alcohol or other factors beyond the direct exposure, potentially encompassing broader maternal psychosocial or behavioral characteristics.

## Introduction

Prenatal alcohol exposure is a widely recognized risk factor for adverse developmental child outcomes, including cognitive, behavioral, and emotional problems [1]. Despite recommendations to abstain from alcohol during pregnancy, some alcohol use among pregnant women remains prevalent [2]. Maternal alcohol consumption has been linked to a direct biological effect on fetal development, with high levels of alcohol consumption linked to fetal alcohol spectrum disorders [3, 4]. On the other hand, paternal alcohol consumption likely influences children through indirect mechanisms, including its impact on the prenatal environment, parental relationships, and household functioning [5]. Men who drink alcohol could create stressful home environments, which can negatively impact the mental health of pregnant women as well as later disrupt the child’s emotional and psychological development. Alcohol misuse in fathers is linked to their mental health problems like depression and anxiety, possibly impairing their parenting and contributing to inconsistent or reduced involvement [6].

Findings on prenatal alcohol consumption are mixed. Some studies found significant associations across a range of behavioral and emotional problems [7–11], while others reported no clear link [12–14]. Study results may vary due to the timing of outcome assessments, as mental health and behavioral effects in children have only been reported at specific ages, and these effects may evolve over time highlighting the need for longer follow-up periods. Different ages during childhood represent various developmental milestones, for example, age 7 is characterized by emerging self-awareness and early peer challenges, while age 11 brings greater emotional regulation but heightened sensitivity to social dynamics [15]. By age 15, adolescents face intense identity exploration and emotional upheaval, and by 18, cognitive maturity supports more independent decision-making [15]. Additionally, inconsistencies arise whether assessments are made by parents or by the children themselves, leading to variability in findings across studies [16].

The Czech Republic ranks among countries with the highest alcohol consumption [17], yet limited research has been conducted on the impact of prenatal alcohol consumption on children’s development. Capitalizing on a Czech birth cohort, this study aims to investigate the association of both maternal and paternal alcohol consumption during the prenatal period with mental health and behavior of their children, as well as how these associations differ based on who reports the outcome and how they change over time. We conduct this analysis at each time point separately to capture age-specific effects, evaluating associations at distinct stages of child development. We examine both maternal and paternal alcohol use to capture the broader family context, in which alcohol use occurs, to also understand how co-occurring parental behaviors during pregnancy contribute to long-term child development.

## Methods

### Participants

We analyzed data from the Czech arm of the European Longitudinal Study of Pregnancy and Childhood (ELSPAC), a prospective cohort study following children from the prenatal stage through to early adulthood. ELSPAC collects information on children’s and their parent’s health and socio-economic status. This project was initiated by the World Health Organization, and the Czech Republic was one of the six participating countries [18]. The Czech arm of ELSPAC (ELSPAC-CZ) includes 7,589 children [19]. Their mothers were enrolled between the 20th week of pregnancy and childbirth. Out of these, 2,438 mothers consented to medical record use only, leaving 5,151 as the baseline ELSPAC-CZ population [19].

This project was approved by the local Czech ethics committee as well as the international committees [19]. Informed consent from participants was obtained at enrollment and at every timepoint of data collection. This study utilizes information from 12 questionnaires collected over 18 years (Supplementary Table S1); the child’s sex and birth date were extracted from medical charts. The secondary use of all ELSPAC-CZ study data was approved by the (C)ELSPAC Ethics Committee at Masaryk University in Brno, Czech Republic (Ref. No. ELSPAC/EK/1/2014, date 09/17/2014).

### Exposures: parental alcohol consumption

We assessed maternal and paternal alcohol consumption during pregnancy using prenatal and newborn questionnaires. In the prenatal questionnaires, the question on maternal alcohol consumption concerned two time points: the first three months of pregnancy and the period of the fetus’ first movement. The question on paternal alcohol consumption concerned only the first three months of the pregnancy. In the newborn questionnaires, the question on both maternal and paternal consumption concerned, retrospectively, the last two months of the pregnancy.

All questionnaires contained the same question: “*How often did you consume any alcoholic beverages [during the period of interest]?”* with six response options ranging from *never* to *at least 10 drinks a day.* To ensure a sufficient sample size per category and to gain information on the frequency-based alcohol consumption during pregnancy, we created three-level variables. For maternal alcohol consumption, the three levels were as follows: *none* (never reported any alcohol consumption during pregnancy) vs. *once* (reported some alcohol consumption at one-time point during pregnancy) vs. *twice or thrice* (reported some alcohol consumption at two or three-time points). And for paternal alcohol consumption: *none* vs. *once* vs. *twice.* By using frequency rather than the number of drinks consumed, our approach aimed to reduce potential bias related to underreporting alcohol consumption during pregnancy.

### Outcome: mental health and behavior of children

Children’s mental health and behavior were assessed using the Strengths and Difficulties Questionnaire (SDQ), a validated tool comprising four subscales: emotional symptoms, conduct problems, hyperactivity/inattention, and peer relationship problems [20–22]. We used the official Czech translation, previously utilized in other studies [23, 24]. Each subscale has five items scored from 0 to 10; the combined score ranges from 0 to 40, where a higher score indicates more difficulties. The SDQ used in the present study was completed by the mothers when children were aged 7, 11, 15, and 18, and by the children themselves at 11, 15, and 18. For the main analysis, the outcome was the total, continuous SDQ score. The Cronbach alphas for subscales of SDQ were low to moderate, ranging from 0.44 to 0.73, and for the full SDQ were moderate, ranging from 0.74 to 0.80 (Supplementary Table S9).

### Covariates

The confounders were chosen a priori based on previous literature [7, 8] and the directed acyclic graph (Supplementary Figure S1). All covariates were collected from the prenatal questionnaire unless specified otherwise (Supplementary Table S1). In the primary analysis, we also adjusted for the child’s sex.

For maternal alcohol consumption exposure, covariates included: mother’s age at child’s birth (continuous), mother’s education (primary vs. secondary vs. tertiary), mother’s employment (on maternity leave vs. employed/working vs. in preparation for career/student/other/not working), house ownership (yes vs. no), mother’s depressive symptoms assessed with Edinburgh Postnatal Depression Scale (EPDS) [25] (continuous), mother’s stressful life events assessed through self-reported 40 questions [26] (continuous), and mother’s family history of alcoholism (considered both mother’s own and her parents’; yes vs. no).

For paternal exposure: father’s age at child’s birth (continuous), father’s education (primary vs. secondary vs. tertiary), father’s employment (employed/working vs. in preparation for career/student/other/not working), house ownership (yes vs. no), father’s depressive symptoms assessed with EPDS [25] (continuous), father’s stressful life events assessed through self-reported 40 questions [26] (continuous), and father’s family history of alcoholism (considered both father’s own and his parents’; yes vs. no).

### Analytical cohort

From 5,151 families, we excluded those missing complete information on maternal and paternal alcohol consumption (*n* = 2,048), without any information on the outcome at any time point (*n* = 936), where mothers and fathers were cohabiting during pregnancy (*n* = 102), or had twins or triplets (*n* = 52), yielding an analytical sample of 2,013 participants (Supplementary Figure S2).

### Statistical analysis

Descriptive data is presented as mean ± standard deviation (SD), median and interquartile range (IQR) or frequency (*n*, %), where appropriate. The primary analysis included only individuals with complete exposure data at all time points. However, this approach may limit statistical power or introduce bias if the missingness is not completely random. To assess the robustness of our findings, we conducted three sensitivity analyses in a cohort of participants who had at least one maternal and one paternal exposure; this allowed us to increase the analytic sample size and statistical power.

In sensitivity analysis one, missing exposure data were treated as no alcohol consumption. This scenario reflects a conservative assumption where non-response is interpreted as non-use, which may be plausible if participants with low-risk behaviors were less likely to report. Conversely, sensitivity analysis two assumed that missing exposure data represented alcohol consumption. This scenario allows us to assess whether our findings remain consistent even when we assume the highest plausible risk associated with missing data. In sensitivity analysis three, we imputed the missing data using the Multivariable Imputation by Chained Equations (MICE) approach. MICE is a statistical method used to deal with missing data; it involves an iterative process where multiple regression models generate various complete datasets using other observed variables to estimate the missing values [27, 28]. Missing data on covariates were imputed using the MICE method. In all models, we assessed standard assumptions for linear regression, including linearity, independence of residuals, and normality of residuals [29]. For the models including paternal exposure, some deviations from these assumptions were observed. To further assess the robustness of our results, we conducted sensitivity analysis four using logistic regression, with the outcome dichotomized to represent clinically relevant SDQ scores: ≤13 vs ≥14 (mother-reported), and ≤ 15 vs ≥16 (child-reported). These are recommended cut-off points for normal vs.borderline and abnormal SDQ [30].

We conducted three main linear regression models. First, we employed linear regression to estimate B with a 95% confidence interval (CI) for the association between parental alcohol consumption, separately for maternal and paternal alcohol consumption, and the total SDQ score at ages 7, 11, 15, and 18 years (reported by mothers and children). Model 1 was adjusted for the parent’s age and child’s sex; Model 2 contained all covariates as specified above; and Model 3 was fully adjusted, entering both maternal and paternal alcohol consumption and all the covariates. As the child’s sex is not directly associated with the exposure, we conducted sensitivity analysis five where we removed it from the list of the covariates. We conducted sensitivity analysis six where we additionally adjusted for variables such as parents’ smoking, their history of alcoholism, and other substance use.

If an association with total SDQ score was significant, we re-ran the analysis for each subscale as outcome (emotional symptoms, conduct problems, hyperactivity/inattention, and peer relationships problems), adjusting for all covariates as in Model 3. In the end, we assessed effect modification by child’s sex and tested an interaction effect between exposure and child’s sex for the associations that were significant. Due to the exploratory nature of our analysis, we did not correct for multiple testing. All analyses were conducted in R version 4.3.0 (2023-04-21).

## Results

We studied 2,013 parent–child triads (Table 1), most mothers (61%) reported never consuming alcohol during pregnancy, while 21% reported drinking some alcohol once and 18% twice or thrice. In contrast, 7% of fathers reported never drinking, 15% once, and 79% twice. The mean mother-reported total SDQ score was 8.9 (age 7), 10.6 (age 11), 8.0 (age 15), and 6.6 (age 18). The mean child-reported total SDQ score was 13.2 (age 11), 11.9 (age 15), and 11.7 (age 18). The distribution of the subscales is presented in the Supplementary Table S2.

**Table 1.** Characteristics of participants (*n* = 2,013)

Participant’s characteristic		Distribution
Child’s sex, *n* (%)	Female	965 (47.9)
	Missing	0 (0)
Mother’s age	Mean (SD)	25.5 (4.7)
	Median [IQR]	25 [22, 28]
	Missing, *n* (%)	1 (0)
Father’s age	Mean (SD)	28.0 (5.7)
	Median [IQR]	27 [24, 31]
	Missing, *n* (%)	41 (2)
**Parental alcohol consumption**		
Maternal alcohol consumption during pregnancy, *n* (%)	None	1,230 (61.1)
	Once	418 (20.8)
	Twice or thrice	365 (18.1)
Paternal alcohol consumption during pregnancy, *n* (%)	None	135 (6.7)
	Once	298 (14.8)
	Twice	1,580 (78.5)
**Child’s mental health and behavior – total SDQ score**		
7 years (reported by mothers)	Mean (SD)	8.9 (4.8)
	Median [IQR]	8 [5, 12]
	Missing, *n* (%)	165 (8.2)
11 years (reported by mothers)	Mean (SD)	10.6 (4.4)
	Median [IQR]	10 [8, 14]
	Missing, *n* (%)	514 (25.5)
11 years (reported by children)	Mean (SD)	13.2 (4.5)
	Median [IQR]	13 [10, 16]
	Missing, *n* (%)	592 (29.4)
15 years (reported by mothers)	Mean (SD)	8.0 (4.4)
	Median [IQR]	7 [16, 10]
	Missing, *n* (%)	978 (48.6)
15 years (reported by children)	Mean (SD)	11.9 (5.7)
	Median [IQR]	11 [7.5, 16]
	Missing, *n* (%)	1,002 (49.8)
18 years (reported by mothers)	Mean (SD)	6.6 (4.6)
	Median [IQR]	5 [3, 9]
	Missing, *n* (%)	1,191 (59.2)
18 years (reported by children)	Mean (SD)	11.7 (5.4)
	Median [IQR]	11 [8, 15]
	Missing, *n* (%)	1,607 (79.8)
*Other characteristics*		
Mother’s education, *n* (%)	Primary	575 (28.8)
	Secondary	831 (41.6)
	Tertiary	593 (29.7)
	Missing	14 (0.7)
Father’s education, *n* (%)	Primary	769 (38.4)
	Secondary	579 (28.9)
	Tertiary	657 (32.8)
	Missing	8 (0.4)
Mother’s employment, *n* (%)	On maternal leave	537 (28.9)
	Employed/working	1,173 (58.7)
	In preparation for a career/student/other/not working	289 (14.5)
	Missing	14 (0.7)
Father’s employment, *n* (%)	Employed/working	1,842 (91.6)
	In preparation for a career/student/other/not working	170 (8.4)
	Missing	1 (0)
House ownership, *n* (%)	Yes	521 (26.5)
	Missing	47 (2.3)
Mother’s depressive symptoms	Mean (SD)	6.2 (4.4)
	Missing, *n* (%)	41 (2)
Father’s depressive symptoms	Mean (SD)	4.1 (3.5)
	Missing, *n* (%)	43 (2.1)
Mother’s stressful events	Median [IQR]	6 [2, 11]
	Missing, *n* (%)	0 (0)
Father’s stressful events	Median [IQR]	5 [2, 10]
	Missing, *n* (%)	0 (0)
Mother’s family history of alcoholism, *n* (%)	Yes	143 (7.2)
	Missing	13 (0.6)
Father’s family history of alcoholism, *n* (%)	Yes	140 (7)
	Missing	17 (0.8)

*Note:* Categorical variables are reported as counts (n) with proportions of non-missing values (%), where appropriate.

Abbreviations: SD = standard deviation; IQR = interquartile range; SDQ = Strengths and Difficulties Questionnaire.

Table 2 presents the associations of parental alcohol consumption with the mental health and behavior of the children. In the adjusted Model 2, for mother-reported SDQ, we found that children whose mothers reported drinking once had higher levels of total SDQ scores only at age 11 years when compared to those whose mothers never reported drinking. Children whose mothers reported drinking twice or thrice had worse outcomes at ages 7, 11, and 18 years. In the fully adjusted Model 3, the association persisted at ages 7, 11, and 18 years. Contrastingly, for child-reported outcomes, the association was present only at age 11 for the children whose mothers reported drinking twice or thrice, relative to those whose mothers reported never drinking. There was no association between paternal alcohol consumption and the outcome in any age in any model, irrespective of by whom it was reported. Results from Model 1, adjusted only for parent’s age and child’s sex, showed a greater magnitude of associations and additionally presented a significant association at ages 7, 15, and 18 years for children whose mothers reported drinking once compared to those who never reported drinking. The results of the six sensitivity analyses are largely in line with the main results (Supplementary Tables S3–S8).

**Table 2.** Association of parental alcohol consumption with mental health and behavior of the children

			Maternal alcohol consumption		Paternal alcohol consumption	
	SDQ		Once	Twice or thrice	Once	Twice
*Reported by mothers*	*7 years*	*Model 1*	0.7 (0.2, 1.3)**	1.1 (0.5, 1.7)***	0 (−1, 1.1)	0.8 (−0.1, 1.7)
		*Model 2*	0.5 (0, 1)	0.8 (0.2, 1.3)**	0.1 (−0.9, 1.1)	0.7 (−0.2, 1.6)
		*Model 3*	0.4 (−0.1, 0.9)	0.7 (0.1, 1.3)*	−0.1 (−1.1, 0.9)	0.5 (−0.4, 1.3)
	*11 years*	*Model 1*	0.9 (0.3, 1.4)**	0.9 (0.4, 1.5)**	0.6 (−0.4, 1.6)	0.8 (−0.1, 1.7)
		*Model 2*	0.7 (0.2, 1.3)**	0.7 (0.1, 1.2)*	0.6 (−0.4, 1.6)	0.7 (−0.2, 1.6)
		*Model 3*	0.7 (0.2, 1.2)*	0.7 (0.1, 1.2)*	0.4 (−0.6, 1.4)	0.4 (−0.4, 1.3)
	*15 years*	*Model 1*	0.9 (0.2, 1.6)**	0.6 (−0.1, 1.3)	0.9 (−0.4, 2.2)	0.3 (−0.9, 1.4)
		*Model 2*	0.7 (0, 1.3)	0.4 (−0.3, 1)	0.9 (−0.4, 2.3)	0.4 (−0.8, 1.5)
		*Model 3*	0.7 (0, 1.3)	0.5 (−0.2, 1.2)	0.6 (−0.7, 1.9)	−0.1 (−1.3, 1)
	*18 years*	*Model 1*	1.1 (0.3, 1.9)**	1.0 (0.2, 1.9)*	0.7 (−1, 2.4)	0.2 (−1.3, 1.7)
		*Model 2*	0.8 (0, 1.5)	0.8 (0, 1.6)*	0.7 (−0.9, 2.4)	0.2 (−1.3, 1.7)
		*Model 3*	0.8 (0, 1.5)	0.9 (0.1, 1.6)*	0.4 (−1.2, 2.1)	−0.3 (−1.7, 1.2)
*Reported by children*	*11 years*	*Model 1*	0.2 (−0.4, 0.8)	1 (0.3, 1.6)**	0.4 (−0.7, 1.5)	0.5 (−0.4, 1.5)
		*Model 2*	0.2 (−0.4, 0.7)	0.8 (0.2, 1.5)**	0.4 (−0.7, 1.5)	0.5 (−0.4, 1.4)
		*Model 3*	0.1 (−0.5, 0.7)	0.8 (0.2, 1.5)*	0.3 (−0.8, 1.4)	0.3 (−0.7, 1.2)
	*15 years*	*Model 1*	0.7 (−0.2, 1.5)	0.6 (−0.3, 1.5)	−0.1 (−1.8, 1.6	0 (−1.5, 1.4)
		*Model 2*	0.4 (−0.5, 1.3)	0.4 (−0.5, 1.3)	0.1 (−1.6, 1.8)	0.2 (−1.2, 1.7)
		*Model 3*	0.3 (−0.5, 1.2)	0.5 (−0.4, 1.4)	−0.1 (−1.8, 1.6)	−0.1 (−1.6, 1.4)
	*18 years*	*Model 1*	0.4 (−1.0, 1.8)	0 (−1.3, 1.3)	−1 (−3.9, 1.9)	−0.8 (−3.4, 1.8)
		*Model 2*	0.1 (−1.3, 1.6)	0 (−1.3, 1.3)	−0.4 (−3.4, 2.5)	−0.1 (−2.7, 2.6)
		*Model 3*	0.1 (−1.3, 1.5)	−0.1 (−1.4, 1.2)	−0.4 (−3.4, 2.6)	−0.3 (−3, 2.4)

*Note:* p-Value < 0.05 *, p-value < 0.01 **, p-value < 0.001 ***, SDQ = Strengths and Difficulties Questionnaire. Results are derived from linear regression and represent B with 95% confidence intervals. The reference categories for both exposures are no alcohol consumption. Model 1 was adjusted for parent’s age and child’s sex and was ran separately for both maternal and paternal alcohol consumption. Model 2 was adjusted for all covariates (parents’ age, child’s sex, parents’ education, parents’ employment, house ownership, parents’ depressive symptoms, parents’ stressful events, and parents’ family history of alcoholism), and was run separately on both maternal and paternal exposure. Model 3 included both maternal and paternal alcohol consumption and all covariates for both parents.

Table 3 shows the results of the fully adjusted Model 3 ran on specific subscales as the outcomes. At age 7, maternal alcohol consumption was associated with worse outcomes in the conduct problems subscale. At age 11, maternal drinking was associated with worse mother-reported outcomes in the emotional symptoms and conduct problems subscale. When child-reported at 11 years, it was related only to the emotional symptoms subscale. At age 18 years, the association was found in the hyperactivity/inattention subscale. When assessing the effect modification by child’s sex, we found an interaction between maternal alcohol consumption and child’s sex only at age 18. When stratified by sex, maternal alcohol consumption once during pregnancy yielded an effect for girls (B 1.2, 95% CI 0.1, 2.4), but no significant effect for boys (B 0.4, 95% CI -0.7, 1.5). However, maternal alcohol consumption twice or thrice yielded an effect for boys (B 1.8, 95% CI 0.6, 2.9), while none for girls (B 0.0, 95% CI -1.2, 1.2).

**Table 3.** Association of maternal alcohol consumption with specific sub-scales of mental health and behavior of the children

			Maternal alcohol consumption	
	SDQ		Once	Twice or thrice
*Reported by mothers*	*7 years*	*Emotional symptoms*	0.2 (0, 0.4)	0.2 (0, 0.4)
		*Conduct problems*	0.2 (0, 0.4)*	0.2 (0, 0.4)*
		*Hyperactivity/inattention*	0.2 (−0.1, 0.4)	0.2 (−0.1, 0.4)
		*Peer relationship problems*	−0.1 (−0.3, 0.1)	0.1 (−0.1, 0.3)
	*11 years*	*Emotional symptoms*	0.3 (0.1, 0.5)**	0.3 (0, 0.5)*
		*Conduct problems*	0.2 (0, 0.4)*	0.1 (−0.1, 0.2)
		*Hyperactivity/inattention*	0.2 (0, 0.4)	0.2 (0, 0.4)
		*Peer relationship problems*	0.0 (−0.2, 0.2)	0.1 (−0.1, 0.3)
	*18 years*	*Emotional symptoms*	0 (−0.3, 0.3)	0.3 (0, 0.6)
		*Conduct problems*	0.2 (0, 0.4)	0.1 (−0.1, 0.3)
		*Hyperactivity/inattention*	0.5 (0.2, 0.8)***	0.5 (0.2, 0.8)**
		*Peer relationship problems*	0.1 (−0.2, 0.3)	0 (−0.2, 0.3)
*Reported by children*	*11 years*	*Emotional symptoms*	0 (−0.2, 0.3)	0.3 (0.1, 0.6)*
		*Conduct problems*	0.1 (−0.1, 0.2)	0.1 (−0.1, 0.3)
		*Hyperactivity/inattention*	0.1 (−0.1, 0.4)	0.2 (0.0, 0.5)
		*Peer relationship problems*	−0.1 (−0.3, 0.1)	0.2 (0, 0.4)

*Note:* p-Value < 0.05 *, p-value < 0.01 **, p-value < 0.001 ***, SDQ = Strengths and Difficulties Questionnaire. These results come from Model 3 that included both maternal and paternal alcohol consumption and it was adjusted for all covariates (parents’ age, child’s sex, parents’ education, parents’ employment, house ownership, parents’ depressive symptoms, parents’ stressful events, and parents’ family history of alcoholism).

## Discussion

In this study of over 2,000 parent–child triads, we found that maternal alcohol consumption during pregnancy was consistently associated with poorer mental health and behavioral outcomes in children, particularly when reported by mothers. Interestingly, we did not observe a frequency–response relationship, nor did the effect size of these associations change with the age of children. When children self-reported their outcomes, the effects were evident only at the age of 11. These associations persisted even after adjusting for paternal alcohol consumption, which showed no significant relationship with the outcomes. Subscale analyses observed effects across various dimensions, without no specific domain predominating. We did not detect any sex differences.

Our results align with a review of 33 studies that suggest that even low-to-moderate maternal prenatal alcohol use is associated with child’s mental health problems [1]. However, a recent review of 36 papers reported no consistent findings [31]. Studies using SDQ also provided conflicting evidence. Some found no association [12–14], while other studies observed worse SDQ outcomes, particularly with binge drinking [7–11]. One study even indicated that children of abstainer mothers had worse SDQ compared to those of light drinkers [11]. Only one study reported that paternal alcohol dependency was linked to worse SDQ [32], which contrasts with our findings. Inconsistencies in the literature might arise due to various reasons: have primarily focused on outcomes in younger children, using various methodologies to measure exposure (e.g., light drinking, binge drinking)., concentrating on internalizing and externalizing symptoms rather than the individual subscales, or utilizing alternative assessment tools such as the Child Behavior Checklist, which further complicates direct comparison [33]. Our study found no clear pattern in whether prenatal alcohol consumption affects externalizing or internalizing symptoms.

Several factors may explain why maternal, rather than paternal, prenatal alcohol consumption is associated with worse mental health and behavior in children. One key factor can be the direct biological impact of alcohol during pregnancy on brain development. Fetal alcohol spectrum disorder manifests with a range of mental health and behavioral problems [3–5, 34]. The association could also be explained by confounding factors that we did not account for or did not measure perfectly. For example, genetic predisposition to mental disorders may be at play, and children whose mothers consume alcohol might inherit a genetic predisposition for mental health disorders [1, 35–38]. There is no evidence that paternal prenatal alcohol consumption does have a direct pathway to affect fetal development, however, some studies suggest that fathers’ alcohol consumption in the preconception period might have a direct biological effect on the fetal through genetic or epigenetic alterations of the sperm [6, 39]. During pregnancy, paternal alcohol consumption may influence the home environment—potentially contributing to increased stress, conflict, or reduced support for the mother [40]. The broader household context remains important, as stress and instability during pregnancy can interact with maternal alcohol use to compound risks to fetal development [41]. However, the significant association observed with maternal exposure in our study suggests that the direct biological effects of alcohol transmitted through the placenta may be more impactful than these indirect environmental influences.

Our results suggest that maternal prenatal alcohol exposure has a modest and inconsistent effect on emotional symptoms, hyperactivity/inattention, and conduct problems, but not on peer problems. Prior studies have also yielded mixed results. Sandtorv found prenatal alcohol consumption to be associated with all SDQ subscales [42]. Prenatal binge drinking correlated with higher internalizing and externalizing scores [9, 10], whereas other studies reported no association [13, 43]. Findings on conduct problems showed inconsistent results as well, with some studies reporting increased risk and others finding no link [7, 8, 44]. Alvik observed an association between binge drinking and emotional symptoms [8], and one study suggested that prenatal maternal alcohol consumption is associated with poorer peer relationship scores [45]. Hyperactivity is the most frequently studied subscale, often used as a proxy for attention deficit hyperactivity disorder (ADHD). However, the evidence remains inconsistent. Two systematic reviews, one covering 13 studies on ADHD and the other one examining six studies focused on SDQ hyperactivity subscales, reported mixed findings [46, 47].

Our findings showed a consistent association between SDQ only when reported by mothers. Total and subscale scores also differed between reporters, with children generally reporting higher SDQ. It is not clear if children overestimate their difficulties or mothers underestimate them. It is possible that children tend to overreport their symptoms, leading to associations that may not reflect true underlying issues. These discrepancies highlight how perceptions of the exposure’s impact may vary by informant and developmental stage [16]. The stronger and more consistent associations in mother-reported outcomes may reflect their broader perspective on behavioral and emotional changes over time [15]. In contrast, children only reported issues that were significantly associated with maternal drinking at age 11, a stage where self-awareness and emotional insight begin to mature [15]. At ages 15 and 18, children may underreport difficulties due to growing autonomy or social desirability [15]. These differences underscore the value of multi-informant approaches in capturing developmental nuances. The lack of significant findings at age 15, when reported by the mother, may reflect the heightened emotions typical of this developmental stage [15]. While each age point in this study marks a distinct developmental milestone, mid-adolescence is uniquely complex. The intense internal changes and social pressures during this period may dilute or mask the measurable impact of external exposures, suggesting that age 15 represents a more variable and less stable window for detecting consistent effects [15].

Previous literature has highlighted the benefit of using multiple reporters, given different perspectives. Rønning argues that self-reports are more prone to biases – such as wanting to look good in front of others or misjudging the severity of symptoms [48]. Authors also argue that if SDQ is used for screening purposes, at least two reporters should be used [48].

Several limitations should be mentioned. First, the use of a frequency-based measure of alcohol consumption does not capture the number of drinking occasions or the amount consumed per occasion, limiting the exposure precision. Additionally, underreporting due to the stigma associated with alcohol use during pregnancy could have led to an underestimation of the associations. Second, missing data and particularly a high drop-out rate in later years for reporting SDQ, might have introduced selection bias. Third, residual confounding from unmeasured or imperfectly measured factors, such as genetic predisposition, environmental stressors, family dynamics, or other medical comorbidities, might be present. Fourth, the outcomes were not reported by fathers, and it is therefore possible that there could be an association if fathers’ perspective was considered. Fifth, as we took an exploratory approach and did not adjust for multiple tests, we cannot exclude the possibility of false positive findings. Sixth, the Cronbach alphas for the subscales were overall low to moderate, whereas the alphas were overall moderate for the full SDQ. Conduct and peer subscales had the lowest alphas, ranging from 0.44 to 0.58, and emotional and hyperactivity subscales ranged from 0.62 to 0.73. This suggests that the conduct and peer subscales are not appropriate to be used as a screening tool on their own, whereas the full SDQ and emotional and hyperactivity subscales could be more suitable. Nevertheless, this study benefits from exposure data on both parents and child and mother outcome reports across multiple years. Furthermore, it provides a unique insight into a high-consumption country like the Czech Republic [17], where prior research on this specific topic is limited.

To conclude, this study adds to the growing evidence that maternal, and not paternal, alcohol consumption during pregnancy is associated with poorer mental health and behavioral outcomes in children. This study reinforces that there is likely no safe level of prenatal alcohol consumption. Public health interventions should continue focusing on education and supporting women to avoid alcohol consumption during pregnancy.

## Supporting information

10.1192/j.eurpsy.2025.10035.sm001 Mohrová et al. supplementary material Mohrová et al. supplementary material

## Data Availability

The data analyzed in this study are available upon request through the website of the Czech ELSPAC project, https://www.celspac.cz/ (accessed on June 12, 2023).

## Supplementary material

For supplementary material accompanying this paper, visit cambridge.org/EPA. The supplementary material for this article can be found at https://doi.org/10.1192/j.eurpsy.2025.10035.
