# Supplementary material for: Maternal and paternal alcohol consumption in the prenatal period and mental health and behavior of their children until adulthood
Source: Eur Psychiatry. 2025 May 30;68(1):e70. doi: 10.1192/j.eurpsy.2025.10035 (PMC12303776; doi:10.1192/j.eurpsy.2025.10035)
Supplement: Mohrová et al. supplementary material [file S0924933825100357sup001.docx]

**Supplementary material**

Table S1 Data sources

Table S2: Distribution of Strengths and Difficulties Questionnaire (SDQ) sub-scales

Table S3 Results of sensitivity analysis 1

Table S4 Results of sensitivity analysis 2

Table S5 Results of sensitivity analysis 3

Table S6 Results of sensitivity analysis 4

Table S7 Results of sensitivity analysis 5

Table S8 Results of sensitivity analysis 6

Table S9 Cronbach alphas

Figure S1 Directed acyclic graph

Figure S2 Participants flow chart

**Table S1** Data sources

| Questionnaire | Respondent | Time period | Data |
| --- | --- | --- | --- |
| Q1 | Mother | Between the 20th week of pregnancy and the birth of the child | Mother’s education, father’s age*, mother’s employment, father’s employment*, father’s education*, house ownership, mother’s family history of alcoholism |
| Q2 | Mother | Between the 20th week of pregnancy and the birth of the child | Maternal alcohol consumption during the first three months of pregnancy and around the period of fetus’ first movement, mother’s stressful life events, mother’s EPDS score |
| Q3 | Father | Between the 20th week of pregnancy and the birth of the child | Paternal alcohol consumption during the first three months of pregnancy, father’s age, father’s education, father’s employment, father’s family history of alcoholism, father’s stressful life events, father’s EPDS score |
| Q4 | Mother | After the birth of the child | Maternal alcohol consumption during the last two months of pregnancy |
| Q5 | Father | after the birth of the child | Paternal alcohol consumption during the last two months of pregnancy |
| Q6 | Mother | 7 years of child | SDQ |
| Q7 | Mother | 11 years of child | SDQ |
| Q8 | Child | 11 years of child | SDQ |
| Q9 | Mother | 15 years of child | SDQ |
| Q10 | Child | 15 years of child | SDQ |
| Q11 | Mother | 18 years of child | SDQ |
| Q12 | Child | 18 years of child | SDQ |

*Note: * used when variable was missing when reported by father*, *EPDS = Edinburgh Postnatal Depression Scale,* *SDQ = Strengths and Difficulties Questionnaire*

**Table S2** Distribution of Strengths and Difficulties Questionnaire (SDQ) sub-scales

| SDQ 7 | Emotional problems sub-scale | Mean (SD) | 1.8 (1.8) |
| --- | --- | --- | --- |
|  |  | Median [IQR] | 1 [0, 3] |
|  |  | Missing, n | 144 |
|  | Conduct sub-scale | Mean (SD) | 1.8 (1.5) |
|  |  | Median [IQR] | 2 [1, 3] |
|  |  | Missing, n | 137 |
|  | Hyperactivity sub-scale | Mean (SD) | 3.5 (2.2) |
|  |  | Median [IQR] | 3 [2, 5] |
|  |  | Missing, n | 141 |
|  | Peer problems sub-scale | Mean (SD) | 1.7 (1.5) |
|  |  | Median [IQR] | 1 [1, 3] |
|  |  | Missing, n | 156 |
| SDQ 11 mother | Emotional problems sub-scale | Mean (SD) | 2.3 (1.6) |
|  |  | Median [IQR] | 2 [1, 3] |
|  |  | Missing, n | 495 |
|  | Conduct sub-scale | Mean (SD) | 2.1 (1.3) |
|  |  | Median [IQR] | 2 [1, 3] |
|  |  | Missing, n | 495 |
|  | Hyperactivity sub-scale | Mean (SD) | 3.6 (1.8) |
|  |  | Median [IQR] | 3 [2, 5] |
|  |  | Missing, n | 495 |
|  | Peer problems sub-scale | Mean (SD) | 2.6 (1.5) |
|  |  | Median [IQR] | 3 [1, 4] |
|  |  | Missing, n | 514 |
| SDQ 15 mother | Emotional problems sub-scale | Mean (SD) | 1.9 (1.8) |
|  |  | Median [IQR] | 1 [0, 3] |
|  |  | Missing, n | 969 |
|  | Conduct sub-scale | Mean (SD) | 1.5 (1.4) |
|  |  | Median [IQR] | 1 [1, 2] |
|  |  | Missing, n | 969 |
|  | Hyperactivity sub-scale | Mean (SD) | 3.3 (1.7) |
|  |  | Median [IQR] | 3 [2, 4] |
|  |  | Missing, n | 977 |
|  | Peer problems sub-scale | Mean (SD) | 1.3 (1.5) |
|  |  | Median [IQR] | 1 [0, 2] |
|  |  | Missing, n | 976 |
| SDQ 18 mother | Emotional problems sub-scale | Mean (SD) | 1.8 (1.8) |
|  |  | Median [IQR] | 1 [0, 3] |
|  |  | Missing, n | 1,191 |
|  | Conduct sub-scale | Mean (SD) | 1.3 (1.3) |
|  |  | Median [IQR] | 1 [0, 2] |
|  |  | Missing, n | 1,191 |
|  | Hyperactivity sub-scale | Mean (SD) | 2.3 (1.8) |
|  |  | Median [IQR] | 2 [1, 3] |
|  |  | Missing, n | 1,189 |
|  | Peer problems sub-scale | Mean (SD) | 1.2 (1.4) |
|  |  | Median [IQR] | 1 [0, 2] |
|  |  | Missing, n | 1,189 |
| SDQ 11 child | Emotional problems sub-scale | Mean (SD) | 3.4 (1.8) |
|  |  | Median [IQR] | 3 [2, 5] |
|  |  | Missing, n | 561 |
|  | Conduct sub-scale | Mean (SD) | 2.8 (1.5) |
|  |  | Median [IQR] | 3 [2, 4] |
|  |  | Missing, n | 533 |
|  | Hyperactivity sub-scale | Mean (SD) | 4.2 (1.8) |
|  |  | Median [IQR] | 4 [3, 5] |
|  |  | Missing, n | 557 |
|  | Peer problems sub-scale | Mean (SD) | 2.8 (1.6) |
|  |  | Median [IQR] | 3 [2, 4] |
|  |  | Missing, n | 534 |

*Note: SDQ = Strengths and Difficulties Questionnaire, SD=standard deviation, IQR=interquartile range*

**Table S3** Results of sensitivity analysis 1

|  |  |  | ***Maternal alcohol consumption*** | | ***Paternal alcohol consumption*** | | |
| --- | --- | --- | --- | --- | --- | --- | --- |
|  | *SDQ* |  | *Once* | *Twice or thrice* | | *Once* | *Twice* |
| *Reported by mothers* | *7 years* | *Model 1* | 0.7 (0.2, 1.2)** | 1.1 (0.6, 1.7)*** | | 0.2 (-0.6, 0.9) | 0.3 (-0.4, 1) |
|  |  | *Model 2* | 0.5 (0, 1)* | 0.8 (0.2, 1.3)** | | 0.1 (-0.7, 0.8) | 0.1 (-0.6, 0.8) |
|  |  | *Model 3* | 0.5 (0, 1) | 0.8 (0.2, 1.3)** | | 0 (-0.7, 0.7) | 0.1 (-0.6, 0.8) |
|  | *11 years* | *Model 1* | 0.8 (0.3, 1.3)** | 1.0 (0.5, 1.5)*** | | 0.4 (-0.4, 1.1) | 0.4 (-0.3, 1) |
|  |  | *Model 2* | 0.7 (0.2, 1.2)** | 0.7 (0.2, 1.2)** | | 0.3 (-0.5, 1) | 0.2 (-0.5, 0.8) |
|  |  | *Model 3* | 0.7 (0.2, 1.2)** | 0.7 (0.2, 1.2)** | | 0.3 (-0.5, 1) | 0.2 (-0.5, 0.9) |
|  | *15 years* | *Model 1* | 0.6 (0, 1.2)* | 0.5 (-0.1, 1.2) | | 0.7 (-0.4, 1.7) | 0.2 (-0.8, 1.1) |
|  |  | *Model 2* | 0.4 (-0.2, 1.1) | 0.4 (-0.2, 1) | | 0.6 (-0.4, 1.7) | 0.2 (-0.8, 1.1) |
|  |  | *Model 3* | 0.4 (-0.2, 1) | 0.5 (-0.1, 1.1) | | 0.5 (-0.5, 1.5) | 0 (-0.9, 0.9) |
|  | *18 years* | *Model 1* | 0.7 (0, 1.5) | 0.8 (0.1, 1.6)* | | 0.7 (-0.6, 2) | -0.2 (-1.4, 1) |
|  |  | *Model 2* | 0.5 (-0.2, 1.2) | 0.6 (-0.1, 1.4) | | 0.5 (-0.7, 1.8) | -0.3 (-1.5, 0.8) |
|  |  | *Model 3* | 0.6 (-0.1, 1.3) | 0.8 (0, 1.5)* | | 0.4 (-0.8, 1.7) | -0.5 (-1.7, 0.7) |
| *Reported by children* | *11 years* | *Model 1* | 0.3 (-0.3, 0.8) | 0.7 (0.2, 1.3)* | | 0.1 (-0.7, 0.9) | 0.3 (-0.4, 1.1) |
|  |  | *Model 2* | 0.2 (-0.3, 0.8) | 0.6 (0, 1.2)* | | 0.1 (-0.7, 0.9) | 0.3 (-0.5, 1) |
|  |  | *Model 3* | 0.2 (-0.3, 0.7) | 0.6 (0, 1.2)* | | 0.1 (-0.7, 0.9) | 0.3 (-0.5, 1) |
|  | *15 years* | *Model 1* | 0.2 (-0.5, 1) | 0.4 (-0.4, 1.2) | | 0.7 (-0.7, 2.0) | 0.2 (-1, 1.4) |
|  |  | *Model 2* | 0 (-0.8, 0.8) | 0.2 (-0.6, 1.1) | | 0.7 (-0.6, 2) | 0.3 (-0.9, 1.5) |
|  |  | *Model 3* | 0 (-0.8, 0.8) | 0.3 (-0.5, 1.1) | | 0.7 (-0.7, 2) | 0.2 (-1, 1.4) |
|  | *18 years* | *Model 1* | 0.1 (-1.2, 1.4) | -0.1 (-1.3, 1.1) | | -1 (-3.2, 1.3) | -1 (-3, 1.1) |
|  |  | *Model 2* | -0.1 (-1.4, 1.2) | -0.1 (-1.3, 1.1) | | -0.9 (-3.1, 1.4) | -1 (-3, 1.1) |
|  |  | *Model 3* | -0.1 (-1.4, 1.2) | -0.1 (-1.3, 1.1) | | -0.7 (-2.9, 1.6) | -0.9 (-3.0, 1.2) |

*Note: In this sensitivity analysis we considered missing exposure data as not drinking alcohol. The sample size of this cohort was 2,531 participants. P-value < 0.05 *, p-value < 0.01 **, p-value < 0.001 ***, SDQ=Strengths and Difficulties Questionnaire. Results are derived from linear regression and represent B with 95% confidence intervals. The reference categories for both exposures are no alcohol consumption. Model 1 was adjusted for parent’s age and child’s sex and was ran separately for both maternal and paternal alcohol consumption. Model 2 was adjusted for all covariates (parents’ age, child’s sex, parents’ education, parents’ employment, house ownership, parents’ depressive symptoms, parents’ stressful events, and parents’ family history of alcoholism), and was ran separately on both maternal and paternal exposure. Model 3 included both maternal and paternal alcohol consumption and all covariates of both parents.*

**Table S4** Results of sensitivity analysis 2

|  |  |  | ***Maternal alcohol consumption*** | | ***Paternal alcohol consumption*** | |
| --- | --- | --- | --- | --- | --- | --- |
|  | *SDQ* |  | *Once* | *Twice or thrice* | *Once* | *Twice* |
| *Reported by mothers* | *7 years* | *Model 1* | 0.7 (0.3, 1.2)** | 1.3 (0.8, 1.8)*** | -0.3 (-1.3, 0.7) | 0.6 (-0.3, 1.5) |
|  |  | *Model 2* | 0.6 (0.1, 1)* | 0.9 (0.5, 1.4)*** | -0.2 (-1.2, 0.8) | 0.6 (-0.3, 1.4) |
|  |  | *Model 3* | 0.5 (0, 0.9)* | 0.9 (0.4, 1.4)*** | -0.4 (-1.3, 0.6) | 0.3 (-0.6, 1.1) |
|  | *11 years* | *Model 1* | 1 (0.5, 1.4)*** | 0.9 (0.4, 1.4)*** | 0.1 (-0.9, 1.1) | 0.6 (-0.3, 1.4) |
|  |  | *Model 2* | 0.8 (0.3, 1.3)*** | 0.6 (0.1, 1.1)* | 0.1 (-0.9, 1.1) | 0.5 (-0.3, 1.3) |
|  |  | *Model 3* | 0.8 (0.3, 1.2)** | 0.6 (0.1, 1.1)* | -0.1 (-1, 0.8) | 0.2 (-0.6, 1.1) |
|  | *15 years* | *Model 1* | 1 (0.4, 1.6)*** | 0.4 (-0.2, 1) | 0.7 (-0.6, 1.9) | 0.2 (-0.9, 1.3) |
|  |  | *Model 2* | 0.8 (0.2, 1.4)** | 0.2 (-0.4, 0.8) | 0.7 (-0.6, 2) | 0.3 (-0.8, 1.4) |
|  |  | *Model 3* | 0.8 (0.2, 1.4)** | 0.3 (-0.3, 0.9) | 0.4 (-0.8, 1.7) | -0.1 (-1.2, 1) |
|  | *18 years* | *Model 1* | 1.4 (0.7, 2.1)*** | 0.8 (0.1, 1.5)* | 0.6 (-1, 2.3) | 0.3 (-1.2, 1.8) |
|  |  | *Model 2* | 1.1 (0.4, 1.8)** | 0.6 (-0.2, 1.3) | 0.7 (-0.9, 2.4) | 0.3 (-1.2, 1.8) |
|  |  | *Model 3* | 1.1 (0.4, 1.8)** | 0.6 (-0.1, 1.3) | 0.2 (-1.4, 1.9) | -0.2 (-1.7, 1.3) |
| *Reported by children* | *11 years* | *Model 1* | 0.3 (-0.2, 0.8) | 0.6 (0, 1.1)* | 0.3 (-0.8, 1.3) | 0.5 (-0.4, 1.4) |
|  |  | *Model 2* | 0.3 (-0.3, 0.8) | 0.4 (-0.1, 1) | 0.3 (-0.7, 1.3) | 0.5 (-0.4, 1.4) |
|  |  | *Model 3* | 0.2 (-0.3, 0.7) | 0.4 (-0.1, 0.9) | 0.2 (-0.8, 1.2) | 0.3 (-0.6, 1.2) |
|  | *15 years* | *Model 1* | 1 (0.2, 1.7)* | 0.3 (-0.5, 1.1) | -0.5 (-2.1, 1.2) | -0.1 (-1.5, 1.4) |
|  |  | *Model 2* | 0.7 (-0.1, 1.5) | 0 (-0.7, 0.8) | -0.2 (-1.9, 1.4) | 0.2 (-1.2, 1.6) |
|  |  | *Model 3* | 0.7 (-0.1, 1.5) | 0.1 (-0.7, 0.9) | -0.4 (-2, 1.3) | 0 (-1.5, 1.4) |
|  | *18 years* | *Model 1* | 0.9 (-0.4, 2.1) | -0.2 (-1.4, 1) | -0.5 (-3.3, 2.4) | -0.8 (-3.4, 1.8) |
|  |  | *Model 2* | 0.7 (-0.5, 2) | -0.3 (-1.4, 0.9) | 0 (-2.9, 2.9) | -0.3 (-3, 2.4) |
|  |  | *Model 3* | 0.7 (-0.6, 1.9) | -0.3 (-1.4, 0.9) | -0.2 (-3.2, 2.8) | -0.5 (-3.2, 2.2) |

*Note: In this sensitivity analysis we considered missing exposure data as drinking alcohol. The sample size of this cohort was 2,531 participants. P-value < 0.05 *, p-value < 0.01 **, p-value < 0.001 ***, SDQ=Strengths and Difficulties Questionnaire. Results are derived from linear regression and represent B with 95% confidence intervals. The reference categories for both exposures are no alcohol consumption. Model 1 was adjusted for parent’s age and child’s sex and was ran separately for both maternal and paternal alcohol consumption. Model 2 was adjusted for all covariates (parents’ age, child’s sex, parents’ education, parents’ employment, house ownership, parents’ depressive symptoms, parents’ stressful events, and parents’ family history of alcoholism), and was ran separately on both maternal and paternal exposure. Model 3 included both maternal and paternal alcohol consumption and all covariates of both parents*

**Table S5** Results of sensitivity analysis 3

*.*

|  |  |  | ***Maternal alcohol consumption*** | | ***Paternal alcohol consumption*** | |
| --- | --- | --- | --- | --- | --- | --- |
|  | *SDQ* |  | *Once* | *Twice or thrice* | *Once* | *Twice* |
| *Reported by mothers* | *7 years* | *Model 1* | 0.9 (0.4, 1.4)*** | 1.1 (0.6, 1.6)*** | -0.2 (-1.1, 0.7) | 0.6 (-0.2, 1.4) |
|  |  | *Model 2* | 0.6 (0.2, 1.1)** | 0.8 (0.3, 1.3)** | -0.2 (-1.1, 0.7) | 0.4 (-0.4, 1.2) |
|  |  | *Model 3* | 0.6 (0.1, 1.1)* | 0.7 (0.2, 1.2)** | -0.4 (-1.3, 0.5) | 0.2 (-0.6, 1) |
|  | *11 years* | *Model 1* | 1 (0.4, 1.5)*** | 1 (0.5, 1.5)*** | -0.2 (-1.1, 0.7) | 0.5 (-0.3, 1.3) |
|  |  | *Model 2* | 0.8 (0.3, 1.3)** | 0.7 (0.2, 1.2)** | -0.2 (-1.1, 0.7) | 0.3 (-0.5, 1.1) |
|  |  | *Model 3* | 0.8 (0.3, 1.3)** | 0.7 (0.2, 1.2)** | -0.3 (-1.2, 0.6) | 0.1 (-0.7, 0.9) |
|  | *15 years* | *Model 1* | 0.9 (0.2, 1.5)** | 0.5 (-0.1, 1.1) | 0.6 (-0.6, 1.8) | 0.3 (-0.8, 1.3) |
|  |  | *Model 2* | 0.7 (0, 1.3)* | 0.4 (-0.2, 1) | 0.7 (-0.5, 1.9) | 0.4 (-0.7, 1.4) |
|  |  | *Model 3* | 0.7 (0.0, 1.3)* | 0.5 (-0.2, 1.1) | 0.5 (-0.7, 1.7) | 0 (-1, 1.1) |
|  | *18 years* | *Model 1* | 0.9 (0.2, 1.7)* | 0.8 (0.1, 1.6)* | 0.6 (-1, 2.1) | 0 (-1.4, 1.4) |
|  |  | *Model 2* | 0.7 (0, 1.4) | 0.7 (-0.1, 1.4) | 0.7 (-0.9, 2.2) | 0.1 (-1.3, 1.5) |
|  |  | *Model 3* | 0.7 (0, 1.5) | 0.8 (0, 1.5)* | 0.4 (-1.2, 1.9) | -0.4 (-1.7, 1) |
| *Reported by children* | *11 years* | *Model 1* | 0.3 (-0.3, 0.8) | 0.7 (0.2, 1.3)** | 0.1 (-0.9, 1.1) | 0.3 (-0.5, 1.2) |
|  |  | *Model 2* | 0.2 (-0.3, 0.7) | 0.6 (0.1, 1.2)* | 0.1 (-0.8, 1.1) | 0.3 (-0.5, 1.2) |
|  |  | *Model 3* | 0.2 (-0.3, 0.8) | 0.6 (0.1, 1.2)* | 0.1 (-0.9, 1) | 0.2 (-0.7, 1) |
|  | *15 years* | *Model 1* | 0.5 (-0.3, 1.3) | 0.3 (-0.5, 1.2) | 0 (-1.5, 1.6) | 0.3 (-1.1, 1.6) |
|  |  | *Model 2* | 0.2 (-0.6, 1.1) | 0.2 (-0.6, 1) | 0.3 (-1.3, 1.9) | 0.5 (-0.8, 1.9) |
|  |  | *Model 3* | 0.2 (-0.6, 1) | 0.2 (-0.6, 1) | 0.2 (-1.3, 1.8) | 0.4 (-1, 1.8) |
|  | *18 years* | *Model 1* | 0.3 (-1, 1.6) | -0.2 (-1.4, 1) | -2.3 (-4.8, 0.3) | -1.7 (-4, 0.5) |
|  |  | *Model 2* | 0.1 (-1.2, 1.4) | -0.2 (-1.4, 1) | -2.2 (-4.7, 0.4) | -1.5 (-3.9, 0.8) |
|  |  | *Model 3* | 0.1 (-1.2, 1.5) | -0.2 (-1.4, 1) | -1.9 (-4.5, 0.7) | -1.4 (-3.7, 1) |

*Note: In this sensitivity analysis we imputed missing exposure data using multiple imputation by chained equations (MICE). The sample size of this cohort was 2,531 participants. P-value < 0.05 *, p-value < 0.01 **, p-value < 0.001 ***, SDQ=Strengths and Difficulties Questionnaire. Results are derived from linear regression and represent B with 95% confidence intervals. The reference categories for both exposures are no alcohol consumption. Model 1 was adjusted for parent’s age and child’s sex and was ran separately for both maternal and paternal alcohol consumption. Model 2 was adjusted for all covariates (parents’ age, child’s sex, parents’ education, parents’ employment, house ownership, parents’ depressive symptoms, parents’ stressful events, and parents’ family history of alcoholism), and was ran separately on both maternal and paternal exposure. Model 3 included both maternal and paternal alcohol consumption and all covariates of both parents.*

**Table S6** Results of sensitivity analysis 4

|  |  |  | ***Maternal alcohol consumption*** | | ***Paternal alcohol consumption*** | |
| --- | --- | --- | --- | --- | --- | --- |
|  | *SDQ* |  | *Once* | *Twice or thrice* | *Once* | *Twice* |
| *Reported by mothers* | *7 years* | *Model 1* | 1.3 (1.0, 1.7) | 1.6 (1.2, 2.1)** | 0.8 (0.5, 1.5) | 1.0 (0.6, 1.7) |
|  |  | *Model 2* | 1.2 (0.9, 1.6) | 1.4 (1.0, 1.9)* | 0.8 (0.5, 1.5) | 1.0 (0.6, 1.6) |
|  |  | *Model 3* | 1.2 (0.9, 1.6) | 1.4 (1.0, 1.9)* | 0.8 (0.4, 1.4) | 0.8 (0.5, 1.4) |
|  | *11 years* | *Model 1* | 1.6 (1.2, 2.1)** | 1.5 (1.1, 2.0)* | 1.0 (0.6, 1.7) | 1.1 (0.7, 1.8) |
|  |  | *Model 2* | 1.5 (1.1, 2.0)* | 1.3 (1.0, 1.8) | 1.0 (0.6, 1.7) | 1.0 (0.6, 1.7) |
|  |  | *Model 3* | 1.5 (1.1, 2.0)* | 1.3 (1.0, 1.8) | 0.9 (0.5, 1.6) | 0.9 (0.5, 1.5) |
|  | *15 years* | *Model 1* | 1.9 (1.2, 3.1)** | 1.6 (1.0, 2.7)* | 1.5 (0.6, 4.0) | 0.9 (0.4, 2.1) |
|  |  | *Model 2* | 1.7 (1.1, 2.8)* | 1.5 (0.9, 2.4) | 1.6 (0.7, 4.4) | 0.9 (0.4, 2.4) |
|  |  | *Model 3* | 1.8 (1.1, 2.9)* | 1.7 (1.0, 2.9)* | 1.3 (0.5, 3.5) | 0.7 (0.3, 1.7) |
|  | *18 years* | *Model 1* | 2.1 (1.2, 3.7)* | 1.7 (0.9, 3.2) | 1.0 (0.3, 3.8) | 0.8 (0.3, 2.8) |
|  |  | *Model 2* | 1.9 (1.0, 3.4)* | 1.6 (0.8, 3.0) | 1.0 (0.3, 4.0) | 0.8 (0.3, 2.9) |
|  |  | *Model 3* | 2.0 (1.1, 3.6)* | 1.7 (0.9, 3.3) | 0.7 (0.2, 2.8) | 0.6 (0.2, 2.2) |
| *Reported by children* | *11 years* | *Model 1* | 0.9 (0.7, 1.2) | 1.3 (1.0, 1.8) | 1.2 (0.7, 2.1) | 1.2 (0.7, 1.9) |
|  |  | *Model 2* | 0.9 (0.6, 1.2) | 1.3 (0.9, 1.7) | 1.2 (0.7, 2.2) | 1.2 (0.7, 1.9) |
|  |  | *Model 3* | 0.9 (0.6, 1.2) | 1.3 (0.9, 1.7) | 1.2 (0.7, 2.1) | 1.1 (0.7, 1.8) |
|  | *15 years* | *Model 1* | 1.2 (0.9, 1.7) | 1.1 (0.7, 1.6) | 1.0 (0.5, 1.9) | 0.9 (0.5, 1.7) |
|  |  | *Model 2* | 1.2 (0.8, 1.6) | 1.0 (0.7, 1.4) | 1.1 (0.5, 2.2) | 1.0 (0.6, 1.9) |
|  |  | *Model 3* | 1.1 (0.8, 1.7) | 1.0 (0.7, 1.5) | 1.0 (0.5, 2.0) | 0.9 (0.5, 1.7) |
|  | *18 years* | *Model 1* | 1.1 (0.6, 2.1) | 1.0 (0.6, 1.8) | 0.6 (0.2, 2.2) | 0.9 (0.3, 3.1) |
|  |  | *Model 2* | 1.0 (0.5, 1.9) | 1.0 (0.6, 1.8) | 0.7 (0.2, 3.0) | 1.3 (0.4, 4.5) |
|  |  | *Model 3* | 1.1 (0.5, 2.0) | 1.0 (0.5, 1.7) | 0.8 (0.2, 3.4) | 1.2 (0.4, 4.5) |

*Note: In this sensitivity analysis we considered the outcome to be binary (the threshold is 13 or below vs 14 or above when reported by mothers, and 15 or below vs 16 and above when reported by children). The sample size of this cohort was 2,013 participants. P-value < 0.05 *, p-value < 0.01 **, p-value < 0.001 ***, SDQ=Strengths and Difficulties Questionnaire. Results are derived from logistic regression and represent odds ratios with 95% confidence intervals. The outcome is binary, where 0 represents normal SDQ and 1 represents borderline and abnormal SDQ. The reference categories for both exposures are no alcohol consumption. Model 1 was adjusted for parent’s age and child’s sex and was ran separately for both maternal and paternal alcohol consumption. Model 2 was adjusted for all covariates (parents’ age, child’s sex, parents’ education, parents’ employment, house ownership, parents’ depressive symptoms, parents’ stressful events, and parents’ family history of alcoholism), and was ran separately on both maternal and paternal exposure. Model 3 included both maternal and paternal alcohol consumption and all covariates of both parents*

**Table S7** Results of sensitivity analysis 5

|  |  |  | ***Maternal alcohol consumption*** | | ***Paternal alcohol consumption*** | |
| --- | --- | --- | --- | --- | --- | --- |
|  | *SDQ* |  | *Once* | *Twice or thrice* | *Once* | *Twice* |
| *Reported by mothers* | *7 years* | *Model 1* | 0.7 (0.2, 1.3)** | 1.2 (0.6, 1.7)*** | 0.1 (-1.0, 1.1) | 0.8 (-0.1, 1.7) |
|  |  | *Model 2* | 0.5 (-0.1, 1.0) | 0.8 (0.2, 1.4)** | 0.1 (-0.9, 1.2) | 0.8 (-0.1, 1.7) |
|  |  | *Model 3* | 0.4 (-0.1, 0.9) | 0.8 (0.2, 1.3)* | 0.0 (-1.0, 1.0) | 0.5 (-0.4, 1.4) |
|  | *11 years* | *Model 1* | 0.9 (0.4, 1.5)** | 1.0 (0.4, 1.6)** | 0.7 (-0.4, 1.7) | 0.9 (0.0, 1.8)* |
|  |  | *Model 2* | 0.8 (0.2, 1.3)** | 0.7 (0.1, 1.3)* | 0.7 (-0.4, 1.7) | 0.8 (-0.1, 1.7) |
|  |  | *Model 3* | 0.7 (0.2, 1.3)** | 0.7 (0.1, 1.3)* | 0.5 (-0.5, 1.5) | 0.6 (-0.3, 1.4) |
|  | *15 years* | *Model 1* | 0.9 (0.2, 1.6)** | 0.6 (-0.1, 1.3) | 0.9 (-0.4, 2.2) | 0.3 (-0.9, 1.4) |
|  |  | *Model 2* | 0.7 (0.0, 1.3) | 0.4 (-0.3, 1.0) | 1.0 (-0.4, 2.3) | 0.4 (-0.8, 1.5) |
|  |  | *Model 3* | 0.7 (0.0, 1.3) | 0.5 (-0.2, 1.2) | 0.6 (-0.7, 1.9) | -0.1 (-1.3, 1.0) |
|  | *18 years* | *Model 1* | 1.1 (0.3, 1.9)** | 1.1 (0.2, 1.9)* | 0.7 (-0.9, 2.4) | 0.2 (-1.3, 1.7) |
|  |  | *Model 2* | 0.8 (0.0, 1.6)* | 0.8 (0.0, 1.6)* | 0.8 (-0.9, 2.4) | 0.3 (-1.2, 1.7) |
|  |  | *Model 3* | 0.8 (0.0, 1.6)* | 0.9 (0.1, 1.7)* | 0.4 (-1.2, 2.1) | -0.2 (-1.7, 1.2) |
| *Reported by children* | *11 years* | *Model 1* | 0.2 (-0.4, 0.8) | 1.0 (0.3, 1.6)** | 0.4 (-0.6, 1.5) | 0.6 (-0.4, 1.5) |
|  |  | *Model 2* | 0.2 (-0.4, 0.7) | 0.8 (0.2, 1.5)** | 0.5 (-0.6, 1.5) | 0.5 (-0.4, 1.5) |
|  |  | *Model 3* | 0.1 (-0.5, 0.7) | 0.8 (0.2, 1.5)** | 0.3 (-0.7, 1.4) | 0.3 (-0.6, 1.3) |
|  | *15 years* | *Model 1* | 0.6 (-0.2, 1.5) | 0.6 (-0.3, 1.5) | -0.3 (-2.0, 1.4) | -0.2 (-1.7, 1.3) |
|  |  | *Model 2* | 0.4 (-0.5, 1.3) | 0.3 (-0.6, 1.2) | 0.0 (-1.8, 1.7) | 0.1 (-1.4, 1.6) |
|  |  | *Model 3* | 0.3 (-0.6, 1.2) | 0.4 (-0.5, 1.3) | -0.3 (-2.0, 1.5) | -0.3 (-1.8, 1.2) |
|  | *18 years* | *Model 1* | 0.4 (-1.0, 1.9) | -0.1 (-1.4, 1.2) | -1.3 (-4.3, 1.6) | -1.2 (-3.8, 1.4) |
|  |  | *Model 2* | 0.2 (-1.2, 1.6) | -0.1 (-1.4, 1.2) | -0.6 (-3.6, 2.4) | -0.3 (-3.0, 2.4) |
|  |  | *Model 3* | 0.1 (-1.3, 1.6) | -0.2 (-1.5, 1.1) | -0.6 (-3.6, 2.4) | -0.5 (-3.2, 2.2) |

*Note: In this sensitivity analysis we excluded child’s sex variable. P-value < 0.05 *, p-value < 0.01 **, p-value < 0.001 ***, SDQ=Strengths and Difficulties Questionnaire. Results are derived from linear regression and represent B with 95% confidence intervals. The reference categories for both exposures are no alcohol consumption. Model 1 was adjusted for parent’s age and was ran separately for both maternal and paternal alcohol consumption. Model 2 was adjusted for all covariates (parents’ age, parents’ education, parents’ employment, house ownership, parents’ depressive symptoms, parents’ stressful events, and parents’ family history of alcoholism), and was ran separately on both maternal and paternal exposure. Model 3 included both maternal and paternal alcohol consumption and all covariates for both parents.*

**Table S8** Results of sensitivity analysis 6

|  |  |  | ***Maternal alcohol consumption*** | | ***Paternal alcohol consumption*** | |
| --- | --- | --- | --- | --- | --- | --- |
|  | *SDQ* |  | *Once* | *Twice or thrice* | *Once* | *Twice* |
| *Reported by mothers* | *7 years* | *Model 1* | 0.7 (0.2, 1.3)** | 1.1 (0.5, 1.7)*** | 0.0 (-1.0, 1.1) | 0.8 (-0.1, 1.7) |
|  |  | *Model 2* | 0.5 (-0.1, 1.0) | 0.7 (0.1, 1.3)* | 0.0 (-1.0, 1.0) | 0.6 (-0.3, 1.5) |
|  |  | *Model 3* | 0.4 (-0.2, 0.9) | 0.7 (0.1, 1.3)* | -0.2 (-1.2, 0.8) | 0.4 (-0.5, 1.2) |
|  | *11 years* | *Model 1* | 0.9 (0.3, 1.4)** | 0.9 (0.4, 1.5)** | 0.6 (-0.4, 1.6) | 0.8 (-0.1, 1.7) |
|  |  | *Model 2* | 0.8 (0.2, 1.3)** | 0.7 (0.1, 1.3)* | 0.5 (-0.5, 1.6) | 0.6 (-0.2, 1.5) |
|  |  | *Model 3* | 0.7 (0.2, 1.3)* | 0.7 (0.1, 1.2)* | 0.4 (-0.6, 1.4) | 0.4 (-0.4, 1.3) |
|  | *15 years* | *Model 1* | 0.9 (0.2, 1.6)** | 0.6 (-0.1, 1.3) | 0.9 (-0.4, 2.2) | 0.3 (-0.9, 1.4) |
|  |  | *Model 2* | 0.7 (0.0, 1.4)* | 0.4 (-0.3, 1.1) | 1.0 (-0.4, 2.3) | 0.4 (-0.8, 1.6) |
|  |  | *Model 3* | 0.7 (0.0, 1.4)* | 0.5 (-0.2, 1.2) | 0.6 (-0.7, 1.9) | -0.1 (-1.2, 1.0) |
|  | *18 years* | *Model 1* | 1.1 (0.3, 1.9)** | 1.0 (0.2, 1.9)* | 0.7 (-1.0, 2.4) | 0.2 (-1.3, 1.7) |
|  |  | *Model 2* | 0.8 (0.0, 1.5) | 0.8 (0.0, 1.6) | 0.8 (-0.9, 2.5) | 0.3 (-1.2, 1.8) |
|  |  | *Model 3* | 0.8 (0.0, 1.6) | 0.8 (0.0, 1.6)* | 0.4 (-1.2, 2.1) | -0.2 (-1.7, 1.3) |
| *Reported by children* | *11 years* | *Model 1* | 0.2 (-0.4, 0.8) | 1.0 (0.3, 1.6)** | 0.4 (-0.7, 1.5) | 0.5 (-0.4, 1.5) |
|  |  | *Model 2* | 0.2 (-0.4, 0.8) | 0.8 (0.2, 1.5)** | 0.4 (-0.7, 1.5) | 0.4 (-0.5, 1.4) |
|  |  | *Model 3* | 0.1 (-0.5, 0.7) | 0.8 (0.2, 1.4)* | 0.3 (-0.8, 1.4) | 0.2 (-0.7, 1.2) |
|  | *15 years* | *Model 1* | 0.7 (-0.2, 1.5) | 0.6 (-0.3, 1.5) | -0.1 (-1.8, 1.6) | 0.0 (-1.5, 1.4) |
|  |  | *Model 2* | 0.4 (-0.5, 1.2) | 0.3 (-0.6, 1.2) | 0.1 (-1.6, 1.8) | 0.2 (-1.3, 1.7) |
|  |  | *Model 3* | 0.3 (-0.6, 1.2) | 0.4 (-0.5, 1.3) | -0.1 (-1.8, 1.6) | -0.2 (-1.7, 1.4) |
|  | *18 years* | *Model 1* | 0.4 (-1.0, 1.8) | 0.0 (-1.3, 1.3) | -1.0 (-3.9, 1.9) | -0.8 (-3.4, 1.8) |
|  |  | *Model 2* | 0.1 (-1.4, 1.5) | -0.1 (-1.4, 1.2) | -0.4 (-3.4, 2.6) | 0.0 (-2.8, 2.7) |
|  |  | *Model 3* | -0.1 (-1.6, 1.4) | -0.2 (-1.5, 1.1) | -0.3 (-3.3, 2.7) | -0.2 (-2.9, 2.6) |

*Note: In this sensitivity analysis we adjusted Model 2 and Model 3 for additional variables: parent’s smoking, parent’s history of alcoholism, and parent’s other substance use. P-value < 0.05 *, p-value < 0.01 **, p-value < 0.001 ***, SDQ=Strengths and Difficulties Questionnaire. Results are derived from linear regression and represent B with 95% confidence intervals. The reference categories for both exposures are no alcohol consumption. Model 1 was adjusted for parent’s age and child’s sex and was ran separately for both maternal and paternal alcohol consumption. Model 2 was adjusted for all covariates (parents’ age, child’s sex, parents’ education, parents’ employment, house ownership, parents’ depressive symptoms, parents’ stressful events, parents’ family history of alcoholism, parent’s smoking, parent’s history of alcoholism, and parent’s other substance use), and was ran separately on both maternal and paternal exposure. Model 3 included both maternal and paternal alcohol consumption and all covariates for both parents.*

**Table S9** Cronbach alphas

| Time point | Subscale | Cronbach alpha |
| --- | --- | --- |
| *SDQ reported by mothers* | | |
| 7 years old | **SDQ total** | 0.77 |
|  | emotional | 0.62 |
|  | conduct | 0.53 |
|  | hyper | 0.73 |
|  | peer | 0.47 |
| 11 years old | **SDQ total** | 0.78 |
|  | emotional | 0.65 |
|  | conduct | 0.55 |
|  | hyper | 0.73 |
|  | peer | 0.5 |
| 15 years old | **SDQ total** | 0.77 |
|  | emotional | 0.65 |
|  | conduct | 0.54 |
|  | hyper | 0.64 |
|  | peer | 0.58 |
| 18 years old | **SDQ total** | 0.80 |
|  | emotional | 0.64 |
|  | conduct | 0.55 |
|  | hyper | 0.67 |
|  | peer | 0.54 |
| *SDQ reported by children* | | |
| 11 years old | **SDQ total** | 0.74 |
|  | emotional | 0.62 |
|  | conduct | 0.49 |
|  | hyper | 0.65 |
|  | peer | 0.46 |
| 15 years old | **SDQ total** | 0.79 |
|  | emotional | 0.70 |
|  | conduct | 0.54 |
|  | hyper | 0.64 |
|  | peer | 0.52 |
| 18 years old | **SDQ total** | 0.78 |
|  | emotional | 0.66 |
|  | conduct | 0.51 |
|  | hyper | 0.65 |
|  | peer | 0.44 |

*Note: SDQ=Strengths and Difficulties Questionnaire*


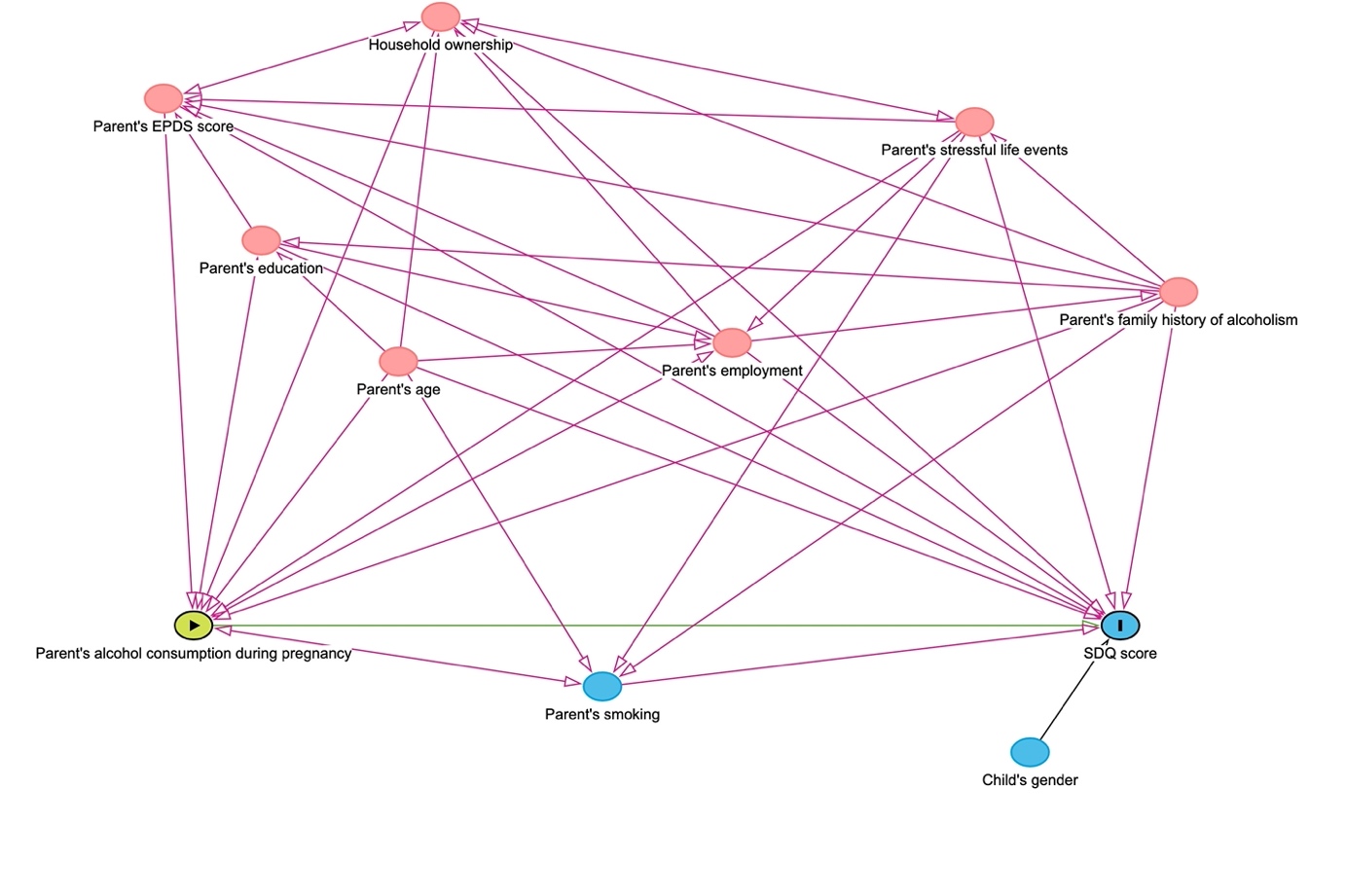


**Figure S1** Directed acyclic graph (DAG).

*Note: Green circle is a exposure, blue circle with I is an outcome, blue circles are ancestors of outcome, and pink circles are ancestors of exposure and outcome. This DAG was created on DAGitty.net.*


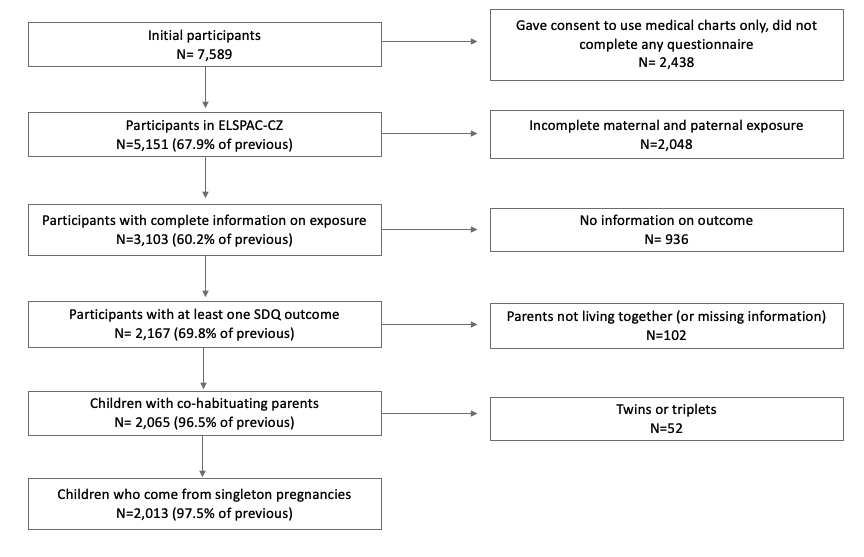


**Figure S2** Participants flow chart
